# Supplementary material for: Usability, User Experience, and Acceptance Evaluation of CAPACITY: A Technological Ecosystem for Remote Follow-Up of Frailty
Source: Sensors (Basel). 2021 Sep 27;21(19):6458. doi: 10.3390/s21196458 (PMC8512153; doi:10.3390/s21196458)

Adapted TAM to the particular use case

- Perceived usefulness:
  - Q1: Using CAPACITY eases remembering my treatment;
  - Q2: Using CAPACITY would improve my independence and autonomy in my daily activities;
  - Q3: Using CAPACITY would increase my daily activity;
  - Q4: Using CAPACITY would enable me controlling my disease;
  - Q5: Using CAPACITY would make easier to commit to my treatment; and
  - Q6: I would find CAPACITY useful to improve my health status.
- Perceived ease of use:
  - Q1: Learning how to use CAPACITY would be easy for me;
  - Q2: It is easy to get CAPACITY to do what I want to do;
  - Q3: Interacting with CAPACITY is clear and understandable;
  - Q4: The interaction with CAPACITY is flexible;
  - Q5: It would be ease for me to become skillful using CAPACITY; and
  - Q6: I would find CAPACITY easy to use.

Ad-hoc acceptance interview

- Q1: The information the device provides motivates me to have a healthier lifestyle;
- Q2: The device makes me feel cared for;
- Q3: Using the device is a burden for me;
- Q4: The device enables me to control my own health; and
- Q5: I would use it.

Tested mobile app and use flows

Gait-speed measurement interaction system and use flow:

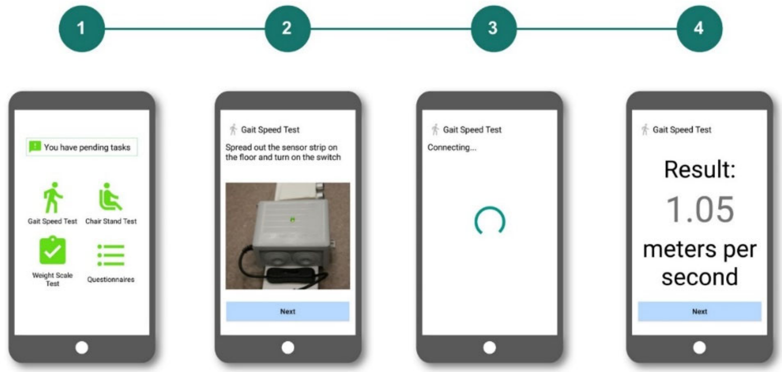

Chair-stand test interaction system and use flow:

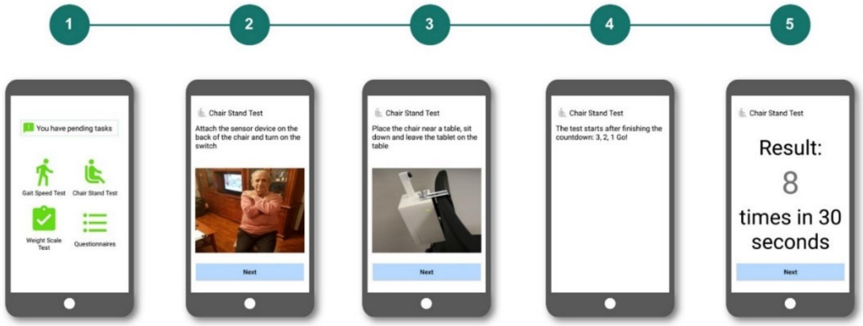

Weight-measurement interaction system and use flow:

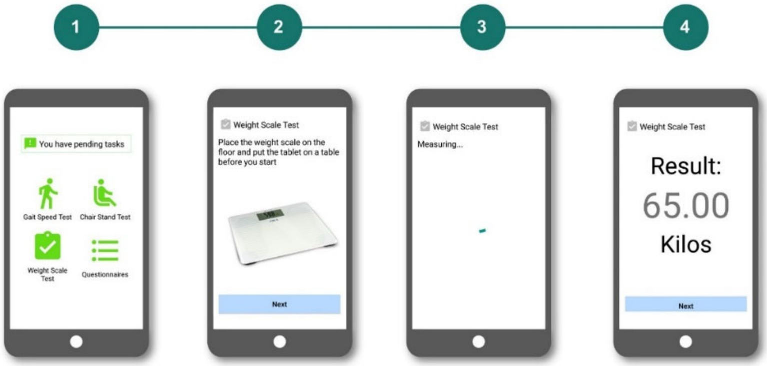

Sample questionnaire (Linda Fried's criteria):

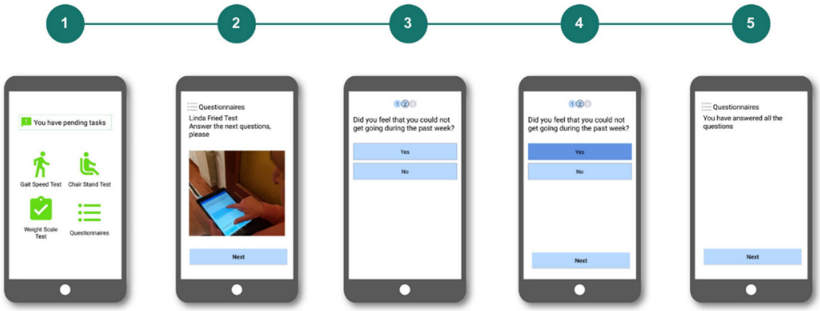

Supplement: Supplementary file 1 [file sensors-21-06458-s001.zip › sensors-1384599-supplementary.pdf]
